# Genetic Variations between Youth and Professional Development Phase English Academy Football Players

**DOI:** 10.3390/genes13112001

**Published:** 2022-11-01

**Authors:** Alexander B.T. McAuley, David C. Hughes, Loukia G. Tsaprouni, Ian Varley, Bruce Suraci, Joseph Baker, Adam J. Herbert, Adam L. Kelly

**Affiliations:** 1 Faculty of Health, Education and Life Sciences, Birmingham City University, Birmingham B15 3TN, UK; 2 Department of Sport Science, Nottingham Trent University, Nottingham NG11 8NS, UK; 3 Academy Coaching Department, AFC Bournemouth, Bournemouth BH7 7AF, UK; 4 School of Kinesiology and Health Science, York University, Toronto, ON M3J 1P3, Canada

**Keywords:** athlete development, genomics, polygenic profile, soccer

## Abstract

The purpose of this study was to examine differences in the genotype frequency distribution of thirty-three single nucleotide variants (SNVs) between youth development phase (YDP) and professional development phase (PDP) academy football players. One hundred and sixty-six male football players from two Category 1 and Category 3 English academies were examined within their specific age phase: YDP (*n* = 92; aged 13.84 ± 1.63 years) and PDP (*n* = 74; aged 18.09 ± 1.51 years). Fisher’s exact tests were used to compare individual genotype frequencies, whereas unweighted and weighted total genotype scores (TGS; TWGS) were computed to assess differences in polygenic profiles. In isolation, the *IL6* (rs1800795) G allele was overrepresented in PDP players (90.5%) compared to YDP players (77.2%; *p* = 0.023), whereby PDP players had nearly three times the odds of possessing a G allele (OR = 2.83, 95% CI: 1.13–7.09). The TGS (*p* = 0.001) and TWGS (*p* < 0.001) were significant, but poor, in distinguishing YDP and PDP players (AUC = 0.643–0.694), with PDP players exhibiting an overall more power-orientated polygenic profile. If validated in larger independent youth football cohorts, these findings may have important implications for future studies examining genetic associations in youth football.

## 1. Introduction

The process of athlete development and ultimately reaching senior professional status in a sport such as football (soccer) is both dynamic and multifactorial [1]. Indeed, task constraints (e.g., the value of deliberate practice and deliberate play, or the importance of early engagement), performer constraints (e.g., differences between skill levels on anthropometric/physiological factors, psychological characteristics, and technical or tactical skill), and environmental constraints (e.g., the influence of birth-place, relative age, and/or socio-cultural influences) have all been associated with the performance of youth football players and their potential to achieve adult success [2]. Despite being heavily researched, the extent to which each of these elements impact performance and affects the likelihood of achieving senior professional status in football remains unclear [3].

The failure to clearly identify a set of variables that uniformly predicts performance levels is, in part, due to methodological issues identified throughout talent identification and development research in football [4]. Prospective and longitudinal analyses in youth football have also revealed that specific performer characteristics may be more important at different time-points throughout development (see [5] for a review). For instance, when comparing English academy football players of different age groups (i.e., under-9 to under-11 vs. under-12 to under-16), Kelly and colleagues found differences in physical characteristics and decision-making [6], technical skill [7], as well as differentiating those who ‘play-up’ an age group [8]. From a longitudinal perspective, Saward et al. [9] performed a ten-year prospective investigation of 2875 male youth football players (aged 8–19 years) from 16 English academies, revealing that future professionals only began to significantly outperform their non-professional counterparts in vertical countermovement jump (CMJ; >0.6 cm) and slalom agility performance (<0.03 s) at the age of 12 years. Moreover, these differences were significantly greater (i.e., >1.7 cm and <0.14 s, respectively) at the age of 18 years, and thus had superior prognostic power.

Although under-researched within a football context, inter-individual genetic variation also appears to influence performance and development in football (see [10] for a review). Moderate to high heritability estimates (i.e., 30–80%) have been reported for anthropometric (e.g., height and skeletal muscle mass = 80%), physiological (e.g., strength and power = 52%), psychological (e.g., personality dimensions and mental toughness = 50%), and technical (e.g., motor control and motor learning = 70%) factors [11,12,13,14,15]. Furthermore, there have been sizeable heritability estimates reported for specific injuries such as anterior cruciate ligament rupture (69%) and overall athlete status (66%) [16,17].

Recent studies have begun to explore which specific genetic variants may explain some of the genetic influence on performance and development in football (e.g., [18,19,20,21]). However, most genetic research in football comprises case-control athlete status designs, which have had limited success [10,22]. Given that the importance of specific characteristics during development in football appears to alter depending on age, the genetic profiles of youth players may also differ between distinct age groups. Indeed, recent research on maturation showed that the genotype frequency distributions of four genetic variants (i.e., *ACTN3* rs1815739, *AGT* rs699, *PPARA* rs4253778, and *NOS3* rs2070744) were significantly different between pre- (aged 10.6 ± 1.4 years) and post- (aged 16.8 ± 2.3 years) peak height velocity academy football players [21].

In England, the structure of football academies is governed by the Premier League’s Elite Player Performance Plan (EPPP) [23], with age groups divided into three development phases, two of which include the youth development phase (YDP; under-12 to under-16) and professional development phase (PDP; under-17 to under-23). The purpose of this study was to examine differences in the genotype frequency distribution, both individually and collectively, of thirty-three single nucleotide variants (SNVs) between YDP and PDP academy football players. These SNVs have been previously associated with physiological (e.g., acceleration and speed), psychological (e.g., personality dimensions and mental toughness), and technical (e.g., dribbling and shooting) phenotypes in academy football players [19,20,21]. Such information may have important implications for future studies examining genetic associations in youth football, as well as advance methodological approaches within this field of research.

## 2. Materials and Methods

### 2.1. Participants

One hundred and sixty-six male football players from two Category 1 and Category 3 English academies participated within their specific age phase: YDP (*n* = 92; aged 13.84 ± 1.63 years) and PDP (*n* = 74; aged 18.09 ± 1.51 years). Informed assent from all players, consent from parents/guardians, and gatekeeper consent from each academy were collected prior to the commencement of the study. All experimental procedures were conducted in accordance with the guidelines in the Declaration of Helsinki and ethical approval was granted by the corresponding author’s institutional Ethics Committee. This study was conducted in accordance with the recommendations for reporting the results of genetic association studies defined by the Strengthening the Reporting of Genetic Association studies (STREGA) statement.

### 2.2. Genetic Procedures

#### 2.2.1. Genotyping

Saliva was collected from players via sterile, self-administered buccal swabs, following a minimum of 30 min since food or drink ingestion. Within 36 h, saliva samples were sent to AKESOgen, Inc. (Peachtree Corners, GA, USA) for DNA extraction. Using Qiagen chemistry, DNA was extracted on an automated Kingfisher FLEX instrument (Thermo Fisher Scientific, Waltham, MA, USA). To measure the quality and quantity of extracted DNA, PicoGreen and Nanodrop measurements were taken. Input to the custom testing array occurs at 200 ng in 20 µL. Amplification, fragmentation, and resuspension were performed using Biomek FXP. GeneTitan instrumentation (Thermo Fisher Scientific, Waltham, MA, USA) was used to stain and scan the arrays, with hybridization performed in a Binder oven at 48 degrees for 24 h, following the Affymetrix Axiom high throughput 2.0 protocol. Data analysis was then performed using raw CEL file data input into the Affymetrix Axiom Analysis Suite (Affymetrix, Santa Clara, CA, USA). Procedures were in accordance with previous studies [19,20,24].

#### 2.2.2. Variant Selection

The SNVs in 33 genes (see Table 1) were selected based on their relevant associations with physiological/injury (i.e., *ACTN3*, *AMPD1*, *ADRB2*, *ACE*, *AGT*, *CPNE5*, *CKM*, *FTO*, *HSD17B14*, *HIF1A*, *IGF1*, *IGF2*, *IL6*, *NOS3*, *PPARA*, *PPARG*, *GALNT13*, *SOD2*, *TRHR*, *UCP2*) and psychological/technical (i.e., *HTR2A*, *BDNF*, *COMT*, *CTNNA2*, *CHRM2*, *DBH*, *DRD1*, *DRD2*, *DRD3*, *DRD4*, *GABRA6*, *OXTR, SLC16A1*) phenotypes in previous studies with academy football players [19,20,21,24,25]. Gene names and symbols are in accordance with those officially approved by the Human Gene Nomenclature Committee (HGNC; https://www.genenames.org). Standard genomic quality control (QC) procedures and thresholds were applied when selecting genetic variants: SNV call rate (>95), sample call rate (>95), Fisher’s linear discriminant (>3.6), and minor allele frequency (>0.05).

#### 2.2.3. Total Genotype Score

Unweighted and weighted total genotype scores (TGS; TWGS) were calculated to assess the differences in polygenic profiles between YDP and PDP players (as described previously [19,20]). Both TGSs and TWGSs have demonstrated sufficient discriminatory power in previous sport genomic research [27,28]. To generate both the TGS and TWGS, each genotype of a respective SNV initially received a score between 0–2 using a data-driven approach based on the observed genotype associations with PDP status. Genotypes of dominant (AA vs. Aa-aa) and recessive (AA-Aa vs. aa) models were assigned a score of two (i.e., associated genotype[s]) or zero (i.e., alternate genotype[s]), whereas genotypes of co-dominant models (AA vs. Aa vs. aa) were assigned three scores (i.e., homozygous-associated genotypes received a score of two, the heterozygote received a score of one, and the alternate homozygous genotype received a score of zero).

For the TGS, the original procedure of Williams and Folland [28] was followed. Genotype scores (GS) were summed and transformed into a 0–100 scale by dividing the total score by the maximum possible score and multiplying by 100.

TGS = (combined − GS/maximum − GS) × 100

For the TWGS, a similar procedure to Varillas Delgado et al. [27] was used. Each GS was multiplied by the β coefficients of each SNV following multiple regression to create weighted genotype scores (WGS). The WGSs were then summed and transformed into a 0–100 scale by dividing the total score by the maximum possible score and multiplying by 100.

TWGS = (combined − WGS/maximum − WGS) × 100

### 2.3. Data Analysis

Data were analyzed using Jamovi version 1.8.1 and IBM SPSS version 25. Fisher’s exact tests were used to test SNVs for adherence with Hardy–Weinberg equilibrium (HWE) and to compare genotype frequencies between YDP and PDP players. Akaike information criterion (AIC) was used to select which genetic model (i.e., co-dominant, dominant, recessive) best fit the data and would be subjected to hypothesis testing. However, if MAF ≤ 0.25, a dominant model was utilized to retain statistical power [21]. An independent *t*-test was used to assess differences in the TGS and TWGS between YDP and PDP players. Additionally, receiver operating characteristic (ROC) curves and area under the curve (AUC) were used to evaluate the discriminatory power of the TGS and TWGS to distinguish YDP and PDP players with threshold values of: >0.5–0.7 = poor, >0.7–0.8 = acceptable, >0.8–0.9 = excellent, and >0.9 = outstanding [29]. Odds ratios (OR) and 95% confidence intervals (CI) were also calculated to estimate the effect size of individual genotypes and polygenic models (split into equal thirds using tertiles). Statistical significance was set at *p* < 0.05.

## 3. Results

**Figure 1:**
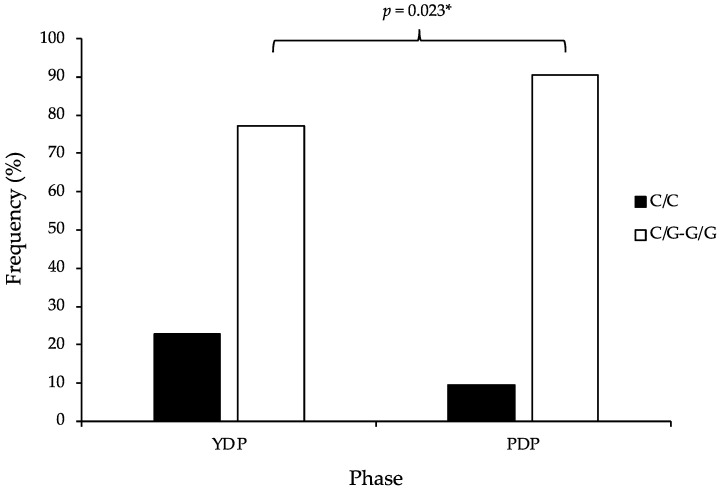
The *IL6* (rs1800795) frequency distribution in youth development phase (YDP) and professional development phase (PDP) English academy football players. * Statistically significant at *p* < 0.05.

The genotype and allele distributions of all SNVs were in HWE, except for *GALNT13* (*p* < 0.001) and *UCP2* (*p* = 0.010) in the PDP group (see Table 2). The genotype frequency distribution of *IL6* was significantly different between YDP and PDP players (*p* = 0.023) (see Figure 1). More specifically, the G allele was overrepresented (13.3%) in PDP players (90.5%) compared to YDP players (77.2%). Furthermore, PDP players had 2.83 times the odds of possessing a G allele (OR = 2.83, 95% CI: 1.13–7.09) compared to YDP players. No significant differences in genotype frequency distribution between the age-specific phases for any other SNVs existed (see Table 3).

**Figure 2:**
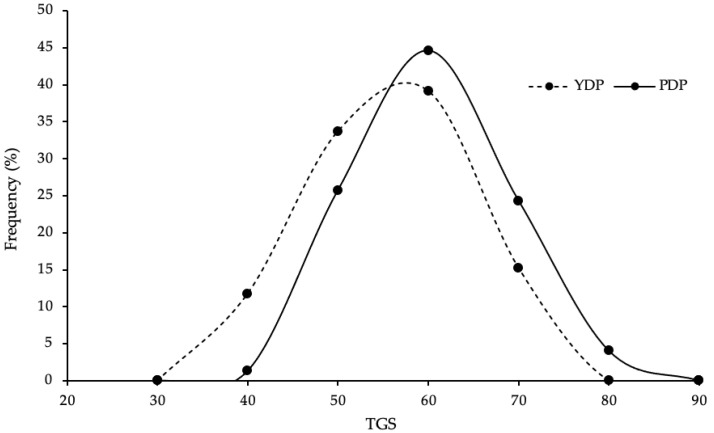
Frequency distribution of total genotype score (TGS) in youth development phase (YDP) and professional development phase (PDP) English academy football players.

The TGS of players ranged from 31 to 69 in the YDP group and 38 to 73 in the PDP group (see Figure 2). The mean TGS of PDP players (54.9 ± 8.41) was significantly higher than YDP players (50.6 ± 8.62; *t* _(164)_ = 3.26, *p* = 0.001). The YDP tertile distribution was: lower = 27, middle = 43, and higher = 22, whereas the PDP tertile distribution was: lower = 16, middle = 24, and higher = 34. Compared to YDP players, PDP players had 2.61 times the odds of having a TGS in the higher third (i.e., 58–73) than a TGS in the lower third (i.e., 31–47; OR = 2.61, CI: 1.15–5.91), as well as 2.77 times the odds of having a TGS in the higher third than a TGS in the middle third (i.e., 48–57; OR = 2.77, CI: 1.33–5.76). The ROC analysis determined that TGS frequency distribution showed significant, but poor, discriminatory power in distinguishing YDP and PDP players (AUC = 0.643, 95% CI: 0.560–0.726).

**Figure 3:**
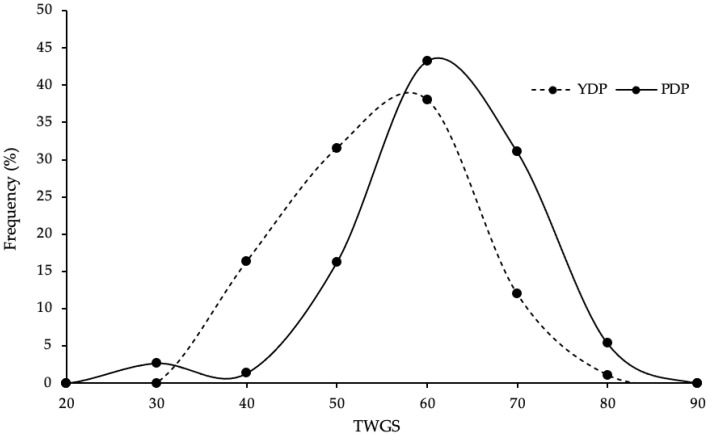
Frequency distribution of total weighted genotype score (TWGS) in youth development phase (YDP) and professional development phase (PDP) English academy football players.

The TWGS of players ranged from 31 to 74 in the YDP group and 24 to 78 in the PDP group (see Figure 3). The mean TWGS of PDP players (56.5 ± 9.63) was significantly higher than YDP players (50.0 ± 9.54; *t* _(164)_ = 4.34, *p* < 0.001). The YDP tertile distribution was: lower = 39, middle = 29, and higher = 24, whereas the PDP tertile distribution was: lower = 11, middle = 24, and higher = 39. Compared to YDP players, PDP players had 5.76 times the odds of having a TWGS in the higher third (i.e., 57–78) than a TWGS in the lower third (i.e., 24–48; OR = 5.76, CI: 2.49–13.35), as well as 2.93 times the odds of having a TWGS in the middle third (i.e., 49–56) than a TWGS in the lower third (OR = 2.93, CI: 1.24–6.94). The ROC analysis determined that TWGS frequency distribution showed significant, but poor, discriminatory power in distinguishing YDP and PDP players (AUC = 0.694, 95% CI: 0.615–0.773).

## 4. Discussion

This study examined differences in the genotype frequency distribution of thirty-three SNVs, both individually and collectively, between YDP and PDP English academy football players. The key findings showed an overrepresentation of the *IL6* (rs1800795) G allele in PDP players compared to YDP players. In addition, the TGS and TWGS models demonstrated that the combination of these thirty-three SNVs was effective in differentiating YDP and PDP players. As such, these results suggest there is significant genetic variation between youth football players of distinct age groups. To our knowledge, this is the first assessment of genotype frequency distribution in isolation, and as part of a polygenic profile, between two age-specific phases of academy football players in England. Therefore, these findings may have important implications for future studies examining genetic associations in youth football.

The *IL6* gene encodes for the pleiotropic cytokine interleukin-6 (IL-6), which has previously been associated with multiple biological processes relevant to sport performance (i.e., glucose homeostasis, muscle hypertrophy, and repairing damaged muscle) [30]. The circulating levels of IL-6 can vary depending on specific variants within the gene. For instance, the G and C alleles of the *IL6* (rs1800795) SNV alter promoter activity and consequently result in higher and lower IL-6 levels, respectively [31]. Higher IL-6 levels have been associated with greater muscle hypertrophy, improved glucose uptake, and increased protection against exercise-induced muscle damage, possibly due to reduced muscle inflammation by positively regulating the pro- and anti-inflammatory cytokine production balance [32,33]. In contrast, lower IL-6 levels may increase the possibility of sustaining a muscular injury, inhibit recovery, and hinder athletic performance, with higher creatine kinase activity reported in response to eccentric exercise in C allele carriers [30].

More recent sport-specific research has shown that IL-6 may be an important biomarker in power-orientated sports and performance phenotypes. Studies assessing Polish and Spanish high performing athletes have reported an overrepresentation of the *IL6* (rs1800795) G allele in those who take part in power-based sports (i.e., jumpers, sprinters, and weightlifters) compared to controls [34,35]. Cross-sectional quantitative data supporting these findings also exist, as youth footballers in Britain possessing the G allele performed significantly better than C allele carriers in acceleration and speed assessments (i.e., 5 m and 20 m sprint) [24]. Therefore, due to the mechanistic properties associated with *IL6* (rs1800795), the G allele may better protect skeletal muscle and aid in repair during powerful muscle contractions, which subsequently allows for a higher volume of training that stimulates favorable adaptations and ultimately results in superior performance in high-intensity activities.

Although power-orientated phenotypes such as acceleration, speed, and vertical jumps are important across all youth football age groups [36], they appear to become more important as players age and mature [37]. For instance, in many male English football academies, youth players do not progress to compete on a full-sized pitch, with eleven players on each team, until the under-13 age group. With this increase in pitch size, players spend more of their competitive match-play time at low speeds and perform a greater number of sprint actions, placing a greater physiological demand on anaerobic capacity [38]. Furthermore, in a longitudinal investigation of English academy football players, it was reported that whilst future professionals began to outperform their non-professional counterparts in vertical CMJ from the age of 12 years (>0.6 cm), differences became more pronounced in older age groups (e.g., aged 18 years > 1.7 cm) [9].

As competitive match-play demands shift more towards anaerobic capacities, academy recruitment teams may choose to retain players displaying superior power rather than endurance capabilities [37]. This may explain the overrepresentation of the *IL6* (rs1800795) G allele in the PDP group compared to the YDP group due to its association with several power-orientated phenotypes. However, recent research in academy football has also shown that the G allele may protect PDP players from injury. More specifically, Hall et al. [25] reported that only post-peak height velocity players (aged 17.5 ± 2.1 years) possessing the *IL6* (rs1800795) C/C genotype suffered significantly more injuries than G allele carriers. The authors noted that the association was possibly due to the combination of greater muscle damage and inflammation experienced by C allele carriers, alongside the higher intensity of match actions and increased frequency of training and/or competitive match-play in older age groups. As such, the overrepresentation of the G allele in the PDP group may be explained by a pleiotropic effect of *IL6* (rs1800795) on power and injury.

The TGS and TWGS models showed that YDP and PDP football players have distinct polygenic profiles, with the TWGS demonstrating greater discriminatory accuracy. This suggests that whilst each SNV has a small additive effect, favorable alleles of individual SNVs have different degrees of influence. This corresponds with previous research in academy football players on physiological, psychological, and technical phenotypes that underpin differences in these age-specific phases [19,20,21]. The general frequency distribution of the genotypes across all SNVs also aligns with the *IL6* (rs1800795) findings. Specifically, PDP players had a greater proportion of alleles previously associated with power-orientated phenotypes (e.g., *ADBR2* rs1042714 G allele, *CKM* rs8111989 T allele, *FTO* rs9939609 A allele, *GALNT13* rs10196189 G allele, *IGF1* rs35767 A allele, *PPARG* rs1801282 G allele, *TRHR* rs7832552 T allele). This indicates PDP players may have an overall more power-orientated polygenic profile, which corresponds with similar findings reported in post-peak height velocity (aged 16.8 ± 2.3 years) academy football players using only four of these SNVs: *ACTN3* (rs1815739), *AGT* (rs699), *PPARA* (rs4253778), and *NOS3* (rs2070744) [21].

The polygenic models also showed that in general YDP players had a greater proportion of favorable alleles in SNVs previously associated with psychological and technical phenotypes (e.g., *HTR2A* rs6311 T allele, *ADBR2* rs1042714 C allele, *BDNF* rs6265 T allele, *DBH* rs1611115 C allele, *DRD1* rs4532 C allele, *DRD4* rs1800955 C allele, *GABRA6* rs3219151 C allele) in academy footballers [19,20]. The importance of these psychological and technical phenotypes in youth football has been demonstrated in previous research by effectively differentiating higher and lower performers in adolescence and predicting success at adulthood [2,3]. However, these findings suggest having an increased frequency of these preferred psychological/technical alleles may be more advantageous in younger age groups. This corresponds with previous research that reported coaches and recruiters consider technical, tactical, and psychological factors as the most important during this stage of development [39,40]. As such, the polygenic models collectively showcase that English academy football players of different age-specific phases may have distinct genetic profiles, with PDP players more power-orientated and YDP players more psychological- and technical-orientated, though further replication studies are required to build on the limited evidence available in youth football players.

Although the polygenic models distinguished YDP and PDP players, they still had relatively poor accuracy, which indicates they should not be considered for practical implementation. Moreover, given the data-driven cross-sectional nature of the analyses, these findings may not generalize well to other youth football cohorts and may reflect cohort effects. Therefore, the external validity of these results should be assessed in larger independent samples alongside the addition of many more relevant genetic variants. It is also important to note that the previous associations of the SNVs included in this study with specific physiological, psychological, technical, and injury phenotypes may not be reliable due to the relatively small sample sizes in football genomic research [10]. Therefore, the inferences made with regards to genetic profile orientation in YDP and PDP players should be interpreted with caution.

Studies with this type of unique sample are typically underpowered so it is important to be relatively conservative with any conclusions, as meaningful implications cannot be made from one study in isolation. However, in the early stages of development in a field, informed speculation based on prior knowledge may be important for informing future work. As a result, we made informed speculation about our findings as a way of guiding subsequent work in this area. Moreover, building this research base with studies using transparent methodologies is important so they can contribute to research synthesis approaches in the future and draw more valid and reliable conclusions before these findings are implemented into applied settings [41].

Nevertheless, this study does have important limitations that should be considered. For instance, we did not make adjustments for multiple comparisons, which may have increased type 1 errors. However, due to the exploratory nature of this study, in regard to the novel experimentation methods employed and the unique cohort, reducing type 2 errors was considered a priority. This is recommended in exploratory research, as a main aim is to ensure an important discovery is not missed in the first instance, which can be validated in subsequent dedicated replication studies [42]. In addition, the sample size (*N* = 166) used in this study was relatively small. However, this was still larger than the median sample size (*N* = 60) reported in a recent review of eighty genetic association studies in football [10]. There were also some deviations from HWE (i.e., *GALNT13* and *UCP2*), which can indicate genotyping error and may have influenced the findings.

## 5. Conclusions

This study has presented novel evidence with regard to the genetic profiles of YDP and PDP male academy football players in England. To be specific, the *IL6* (rs1800795) G allele was overrepresented in PDP players compared to YDP players, possibly due to its theorised pleiotropic effect on power and injury phenotypes. Moreover, the TGS and TWGS models derived from all thirty-three SNVs effectively distinguished YDP and PDP players, with PDP players exhibiting an overall more power-orientated polygenic profile. As such, this study has shown for the first time that there is significant inter-individual genetic variation between youth football players of specific age phases in English academies. If validated in larger independent youth football cohorts, these findings may have important implications for future studies examining genetic associations in youth football.

## Figures and Tables

**Table 1:** Gene and single nucleotide variant (SNV) information.

Gene	Symbol	Chr	SNV	Consequence	MAF
5-hydroxytryptamine receptor 2A	*HTR2A*	13q14.2	rs6311	Intron variant C > T	T = 0.44
Actinin α 3	*ACTN3*	11q13.2	rs1815739	Nonsense variant C > T (Arg > Ter)	T = 0.43
Adenosine monophosphate deaminase 1	*AMPD1*	1p13.2	rs17602729	Nonsense variant G > A (Gln > Ter)	A = 0.12
Adrenoceptor β 2	*ADRB2*	5q32	rs1042714	Missense variant G > C (Glu > Gln)	G = 0.41
Angiotensin I converting enzyme	*ACE*	17q23.3	rs4341	Intron variant C > G (Insertion > Deletion)	C = 0.43
Angiotensinogen	*AGT*	1q42.2	rs699	Missense variant A > G (Met > Thr)	G = 0.41
Brain derived neurotrophic factor	*BDNF*	11p14.1	rs6265	Missense variant C > T (Val > Met)	T = 0.20
Catechol-O-methyltransferase	*COMT*	22q11.21	rs4680	Missense variant G > A (Val > Met)	A = 0.50
Catenin α 2	*CTNNA2*	2p12	rs7600563	Intron variant T > G	G = 0.34
Cholinergic receptor muscarinic 2	*CHRM2*	7q33	rs1824024	Intron variant C > A	C = 0.29
Copine 5	*CPNE5*	6p21.2	rs3213537	Intron variant C > T	T = 0.14
Creatine kinase, M-type	*CKM*	19q13.32	rs8111989	500B Downstream variant T > C	C = 0.30
Dopamine β-hydroxylase	*DBH*	9q34.2	rs1611115	2KB Upstream variant C > T	T = 0.21
Dopamine receptor D1	*DRD1*	5q35.2	rs4532	5 Prime UTR variant C > T	C = 0.40
Dopamine receptor D2	*DRD2*	11q23.2	rs1076560	Intron variant C > A	A = 0.15
Dopamine receptor D3	*DRD3*	3q13.31	rs6280	Missense variant C > T (Gly > Ser)	C = 0.33
Dopamine receptor D4	*DRD4*	11p15.5	rs1800955	2KB Upstream variant T > C	C = 0.41
FTO α-ketoglutarate dependent dioxygenase	*FTO*	16q12.2	rs9939609	Intron variant T > A	A = 0.41
γ-aminobutyric acid type A receptor subunit alpha6	*GABRA6*	5q34	rs3219151	3 Prime UTR variant C > T	C = 0.42
Hydroxysteroid 17-β dehydrogenase 14	*HSD17B14*	19q13.33	rs7247312	Intron variant A > G	G = 0.10
Hypoxia inducible factor 1 subunit α	*HIF1A*	14q23.2	rs11549465	Missense variant C > T (Pro > Ser)	T = 0.10
Insulin-like growth factor 1	*IGF1*	12q23.2	rs35767	Missense variant G > A (Gly > Val)	A = 0.16
Insulin-like growth factor 2	*IGF2*	11p15.5	rs680	3 Prime UTR variant C > T	T = 0.32
Interleukin 6	*IL6*	7p15.3	rs1800795	Intron variant G > C	C = 0.42
Nitric oxide synthase 3	*NOS3*	7q36.1	rs2070744	Intron variant C > T	C = 0.44
Oxytocin receptor	*OXTR*	3p25.3	rs2254295	Intron variant C > T	C = 0.11
Peroxisome proliferator activated receptor α	*PPARA*	22q13.31	rs4253778	Intron variant G > C	C = 0.19
Peroxisome proliferator activated receptor γ	*PPARG*	3p25.2	rs1801282	Missense variant C > G (Pro > Ala)	G = 0.12
Polypeptide N-acetylgalactosaminyltransferase 13	*GALNT13*	2q23.3-q24.1	rs10196189	Intron variant A > G	G = 0.14
Solute carrier family 16 member 1	*SLC16A1*	1p13.2	rs1049434	Missense variant T > A (Asp > Glu)	A = 0.44
Superoxide dismutase 2	*SOD2*	6q25.3	rs4880	Missense variant A > G (Val > Ala)	G = 0.47
Thyrotropin releasing hormone receptor	*TRHR*	8q23.1	rs7832552	Intron variant C > T	T = 0.27
Uncoupling protein 2	*UCP2*	11q13.4	rs660339	Missense variant G > A (Ala > Val)	A = 0.40

Note. Chr = chromosome location; MAF = minor allele frequency (according to European population; 1000 Genomes Project Consortium [26]).

**Table 2:** Descriptive statistics of youth and professional development phase English academy football players.

Gene (SNV)	Genotype	YDP = *n* (%)	PDP = *n* (%)	All = *n* (%)	MAF	HWE
*HTR2A* (rs6311)	C/C	39 (42)	25 (34)	64 (39)	0.40	0.26
	C/T	36 (39)	36 (49)	72 (43)		
	T/T	17 (18)	13 (18)	30 (18)		
*ACE* (rs4341)	G/G	27 (29)	21 (28)	48 (29)	0.47	0.76
	G/C	45 (49)	35 (47)	80 (48)		
	C/C	20 (22)	18 (24)	38 (23)		
*ACTN3* (rs1815739)	C/C	34 (37)	26 (35)	60 (36)	0.39	0.42
	C/T	46 (50)	38 (51)	84 (51)		
	T/T	12 (13)	10 (14)	22 (13)		
*ADBR2* (rs1042714)	C/C	27 (29)	20 (27)	47 (28)	0.48	0.44
	C/G	43 (47)	35 (47)	78 (47)		
	G/G	22 (24)	19 (26)	41 (25)		
*AGT* (rs699)	A/A	26 (28)	24 (32)	50 (30)	0.45	1
	A/G	48 (52)	34 (46)	82 (49)		
	G/G	18 (20)	16 (22)	34 (20)		
*AMPD1* (rs17602729)	G/G	74 (80)	59 (80)	133 (80)	0.11	0.70
	G/A	17 (18)	14 (19)	31 (19)		
	A/A	1 (1)	1 (1)	2 (1)		
*BDNF* (rs6265)	C/C	60 (65)	50 (68)	110 (66)	0.19	1
	C/T	28 (30)	22 (30)	50 (30)		
	T/T	4 (4)	2 (3)	6 (4)		
*COMT* (rs4680)	G/G	30 (33)	22 (31)	53 (32)	0.43	0.87
	G/A	47 (51)	36 (49)	83 (50)		
	A/A	15 (16)	15 (20)	30 (18)		
*CTNNA2* (rs7600563)	T/T	44 (52)	37 (51)	81 (51)	0.28	1
	T/G	34 (40)	31 (42)	65 (41)		
	G/G	7 (8)	5 (7)	12 (8)		
*CHRM2* (rs1824024)	A/A	37 (40)	31 (42)	68 (41)	0.36	0.87
	A/C	42 (46)	36 (49)	78 (47)		
	C/C	13 (14)	7 (9)	20 (12)		
*CPNE5* (rs3213537)	C/C	63 (73)	48 (66)	111 (70)	0.16	0.77
	C/T	21 (25)	24 (33)	45 (28)		
	T/T	2 (2)	1 (1)	3 (2)		
*CKM* (rs8111989)	T/T	45 (49)	41 (55)	86 (52)	0.28	0.85
	T/C	38 (41)	28 (38)	66 (40)		
	C/C	9 (10)	5 (7)	14 (8)		
*DBH* (rs1611115)	C/C	56 (61)	44 (59)	100 (60)	0.22	0.50
	C/T	33 (36)	27 (36)	60 (36)		
	T/T	3 (3)	3 (4)	6 (4)		
*DRD1* (rs4532)	T/T	35 (38)	33 (45)	68 (41)	0.34	0.30
	T/C	46 (50)	36 (49)	82 (49)		
	C/C	11 (12)	5 (7)	16 (10)		
*DRD2* (rs1076560)	C/C	65 (71)	46 (62)	111 (67)	0.19	0.61
	C/A	23 (25)	25 (34)	48 (29)		
	A/A	4 (4)	3 (4)	7 (4)		
*DRD3* (rs6280)	T/T	36 (39)	28 (38)	64 (39)	0.37	0.74
	T/C	42 (46)	38 (51)	80 (48)		
	C/C	14 (15)	8 (11)	22 (13)		
*DRD4* (rs1800955)	C/C	20 (25)	14 (22)	34 (23)	0.44	0.18
	C/T	32 (40)	32 (49)	64 (44)		
	T/T	29 (36)	19 (29)	48 (33)		
*FTO* (rs9939609)	T/T	31 (34)	20 (27)	51 (31)	0.44	0.87
	T/A	41 (45)	43 (58)	84 (51)		
	A/A	20 (22)	11 (15)	31 (19)		
*GABRA6* (rs3219151)	T/T	27 (30)	22 (30)	49 (30)	0.44	0.35
	T/C	47 (52)	40 (55)	87 (53)		
	C/C	17 (19)	11 (15)	28 (17)		
*GALNT13* (rs10196189)	A/A	63 (68)	46 (62)	109 (66)	0.22	<0.001
	A/G	23 (25)	18 (24)	41 (24)		
	G/G	6 (7)	10 (14)	16 (10)		
*HIF1A* (rs11549465)	C/C	69 (75)	57 (77)	126 (76)	0.13	1
	C/T	22 (24)	16 (22)	38 (23)		
	T/T	1 (1)	1 (1)	2 (1)		
*HSD17B14* (rs7247312)	A/A	72 (78)	62 (84)	134 (81)	0.11	0.39
	A/G	17 (18)	12 (16)	29 (17)		
	G/G	3 (3)	0 (0)	3 (2)		
*IGF1* (rs35767)	G/G	65 (71)	44 (59)	109 (66)	0.18	0.60
	G/A	26 (28)	27 (36)	53 (32)		
	A/A	1 (1)	3 (4)	4 (2)		
*IGF2* (rs680)	C/C	49 (53)	35 (47)	84 (51)	0.28	0.34
	C/T	37 (40)	35 (47)	72 (43)		
	T/T	6 (7)	4 (6)	10 (6)		
*IL6* (rs1800795)	G/G	32 (35)	29 (39)	61 (37)	0.40	0.75
	G/C	39 (42)	38 (51)	77 (46)		
	C/C	21 (23)	7 (9)	28 (17)		
*NOS3* (rs2070744)	T/T	37 (40)	28 (38)	65 (39)	0.36	0.41
	T/C	42 (46)	40 (54)	82 (49)		
	C/C	13 (14)	6 (8)	19 (11)		
*OXTR* (rs2254295)	T/T	68 (79)	57 (78)	125 (79)	0.12	0.06
	T/C	15 (17)	14 (19)	29 (18)		
	C/C	3 (3)	2 (3)	5 (3)		
*PPARA* (rs4253778)	G/G	55 (60)	52 (70)	107 (65)	0.20	0.34
	G/C	33 (36)	17 (23)	50 (30)		
	C/C	4 (4)	5 (7)	9 (5)		
*PPARG* (rs1801282)	C/C	77 (84)	60 (81)	137 (83)	0.09	0.64
	C/G	15 (16)	12 (16)	27 (16)		
	G/G	0 (0)	2 (3)	1 (1)		
*SLC16A1* (rs1049434)	T/T	32 (35)	23 (31)	55 (33)	0.42	0.87
	T/A	46 (50)	37 (50)	83 (50)		
	A/A	14 (15)	14 (19)	28 (17)		
*SOD2* (rs4880)	A/A	26 (28)	19 (26)	45 (27)	0.49	0.64
	A/G	40 (43)	40 (54)	80 (48)		
	G/G	26 (28)	15 (20	41 (25)		
*TRHR* (rs7832552)	C/C	51 (55)	38 (51)	89 (54)	0.29	0.09
	C/T	30 (33)	29 (39)	59 (36)		
	T/T	11 (12)	7 (9)	18 (11)		
*UCP2* (rs660339)	G/G	27 (29)	17 (23)	44 (27)	0.44	0.03
	G/A	49 (53)	48 (65)	97 (58)		
	A/A	16 (17)	9 (12)	25 (15)		

Note. YDP = youth development phase; PDP = professional development phase; MAF = minor allele frequency; HWE = Hardy–Weinberg equilibrium.

**Table 3:** Genetic associations with youth and professional development phase English academy footballers.

Gene (SNV)	Model	YDP (%)	PDP (%)	*B*	OR (95% CI)	*p*
*HTR2A* (rs6311)	C/C	42	34	1.35	0.69 (0.37–1.31)	0.260
	C/T-T/T	58	66			
*ACE* (rs4341)	G/G	29	28	0.31	0.95 (0.49–1.88)	1
	G/C-C/C	71	72			
*ACTN3* (rs1815739)	C/C	37	35	0.28	0.92 (0.49–1.75)	0.871
	C/T-T/T	63	65			
*ADBR2* (rs1042714)	C/C	29	27	0.24	0.89 (0.45–1.76)	0.863
	C/G-G/G	71	73			
*AGT* (rs699)	A/A	28	32	0.74	1.22 (0.63–2.37)	0.611
	A/G-G/G	72	68			
*AMPD1* (rs17602729)	G/G	80	80	0.37	0.96 (0.44–2.06)	1
	G/A-A/A	20	20			
*BDNF* (rs6265)	C/C	65	68	0.11	1.11 (0.58–2.13)	0.869
	C/T-T/T	35	32			
*COMT* (rs4680)	G/G-G/A	84	80	1.57	0.77 (0.35–1.69)	0.547
	A/A	16	20			
*CTNNA2* (rs7600563)	T/T	52	51	0.42	0.96 (0.51–1.79)	1
	T/G-G/G	48	49			
*CHRM2* (rs1824024)	A/A	40	42	0.25	1.07 (0.58–2.00)	0.875
	A/C-C/C	60	58			
*CPNE5* (rs3213537)	C/C	73	66	0.40	0.70 (0.36–1.38)	0.386
	C/T-T/T	27	34			
*CKM* (rs8111989)	T/T	49	55	0.72	1.30 (0.70–2.40)	0.437
	T/C-C/C	51	45			
*DBH* (rs1611115)	C/C	61	60	0.03	0.94 (0.50–1.76)	0.874
	C/T-T/T	39	40			
*DRD1* (rs4532)	T/T	38	45	0.02	1.31 (0.70–2.44)	0.430
	T/C-C/C	62	55			
*DRD2* (rs1076560)	C/C	71	62	1.26	0.68 (0.36–1.31)	0.320
	C/A-A/A	29	38			
*DRD3* (rs6280)	T/T	39	38	0.32	0.95 (0.50–1.78)	0.874
	T/C-C/C	61	62			
*DRD4* (rs1800955)	C/C	25	22	0.36	0.84 (0.38–1.82)	0.697
	C/T-T/T	75	78			
*FTO* (rs9939609)	T/T	34	27	0.93	0.73 (0.37–1.43)	0.400
	T/A-A/A	66	73			
*GABRA6* (rs3219151)	T/T-T/C	81	85	0.33	1.29 (0.56–2.97)	0.677
	C/C	19	15			
*GALNT13* (rs10196189)	A/A	68	62	0.01	0.76 (0.40–1.44)	0.415
	A/G-G/G	32	38			
*HIF1A* (rs11549465)	C/C	75	77	0.13	1.12 (0.54–2.29)	0.856
	C/T-T/T	25	23			
*HSD17B14* (rs7247312)	A/A	78	84	0.63	1.44 (0.65–3.17)	0.431
	A/G-G/G	22	16			
*IGF1* (rs35767)	G/G	71	59	1.34	0.61 (0.32–1.16)	0.142
	G/A-A/A	29	41			
*IGF2* (rs680)	C/C	53	47	0.94	0.79 (0.43–1.45)	0.532
	C/T-T/T	47	53			
*IL6* (rs1800795)	G/G-G/C	77	91	3.00	2.83 (1.13–7.09)	0.023 *
	C/C	23	9			
*NOS3* (rs2070744)	T/T	40	38	0.21	0.90 (0.48–1.70)	0.873
	T/C-C/C	60	62			
*OXTR* (rs2254295)	T/T	79	78	0.07	0.94 (0.44–2.02)	1
	T/C-C/C	21	22			
*PPARA* (rs4253778)	G/G	60	70	0.00	1.59 (0.83–3.05)	0.193
	G/C-C/C	40	30			
*PPARG* (rs1801282)	C/C	84	81	1.49	0.83 (0.37–1.86)	0.685
	C/G-G/G	16	19			
*SLC16A1* (rs1049434)	T/T	35	31	0.68	0.85 (0.44–1.62)	0.624
	T/A-A/A	65	69			
*SOD2* (rs4880)	A/A-A/G	72	80	0.64	1.55 (0.75–3.20)	0.279
	G/G	28	20			
*TRHR* (rs7832552)	C/C	55	51	0.11	0.85 (0.46–1.57)	0.640
	C/T-T/T	45	49			
*UCP2* (rs660339)	G/G	29	23	0.48	0.72 (0.36–1.45)	0.381
	G/A-A/A	71	77			

Note. Bold values and * highlight statistical significance at *p* < 0.05. YDP = youth development phase; PDP = professional development phase; *B* = unstandardized β; OR = odds ratio; CI = confidence interval.

## Data Availability

The data presented in this study are available on request from the corresponding author.
